# CD74 interacts with APP and suppresses the production of Aβ

**DOI:** 10.1186/1750-1326-4-41

**Published:** 2009-10-22

**Authors:** Shuji Matsuda, Yukiko Matsuda, Luciano D'Adamio

**Affiliations:** 1 Albert Einstein College of Medicine, Department of Microbiology & Immunology, 1300 Morris Park Avenue, Bronx, NY 10461, USA

## Abstract

**Background:**

Alzheimer disease (AD) is characterized by senile plaques, which are mainly composed of β amyloid (Aβ) peptides. Aβ is cleaved off from amyloid precursor protein (APP) with consecutive proteolytic processing by β-secretase and γ-secretase.

**Results:**

Here, we show that CD74, the invariant chain of class II major histocompatibility complex, interacts with APP and serves as a negative regulator of Aβ. CD74 resembles other APP interacters such as BRI2 and BRI3, since all of them reduce the level of Aβ. However, unlike BRIs, CD74 does not reduce the secretion of sAPPα or sAPPβ. Interestingly, in HeLa cells, over expression of CD74 steers APP, but not Notch, to large vacuoles created by CD74.

**Conclusion:**

Taken together, we propose that CD74 inhibits Aβ production by interacting with and derailing normal trafficking of APP.

## Introduction

About 1% of humans aged 60-64 years have AD, increasing steadily to as many as 35%-40% after age 85 [1]. AD progressively leads to a severely impaired state and complete social dependence. At autopsy, cerebral atrophy, neurofibrillary tangles and amyloid plaques are observed in the hippocampus, entorhinal cortex, amygdala and other areas. Tangles consist of intraneuronal masses of helically wound filaments of the hyperphosphorylated protein, tau. Plaques are extracellular deposits of Aβ, a peptide derived from cleavage of APP, surrounded by dystrophic neurites. Most AD cases present Aβ deposits in cortical and/or meningeal micro vessels. In a minority of cases, this vascular cerebral amyloid angiopathy (CAA) is rather severe [2].

APP is a type I transmembrane protein that undergoes a series of proteolytic cleavages [3]. β-secretase cleaves APP into a soluble ectodomain (sAPPβ) and a membrane bound C-terminal fragment of 99 amino acids (C99). C99 is cleaved by the γ-secretase, which consists of a multicomponent complex comprised of Presenilins (PS1 and PS2), Nicastrin (NCT), PEN2 and APH1 [4]. The γ-cleavage releases two peptides: Aβ peptide, consisting of 2 major species of 40 and 42 amino acids (Aβ40 and Aβ42, respectively) and an intracellular product AID/AICD, Several evidence point to the short AID/AICD as a biologically active intracellular peptide, which may modulate cell death, Notch signaling, gene transcription and Ca^++ ^homeostasis [5-19]. In an alternative pathway, APP is processed by α-secretase within the Aβ sequence, leading to the production of the soluble sAPPα ectodomain and a membrane bound C-terminal fragment of 83 amino acids (C83). After age, a family history of dementia is major risk factor for AD and 10%-15%, of all AD subjects have a family history consistent with an autosomal dominant trait. These familial cases (FAD) are due to mutations in APP and in Presenilins because they alter the rate of APP processing and Aβ42 generation.

Given the role of APP processing by secretases to AD pathology and APP-mediated functions, identifying the molecules that regulate APP cleavage is physiologically relevant and of therapeutic interest. A genetic screen aimed to the identification of regulators of APP processing led to the identification of BRI2 and BRI3 as APP ligands that inhibit Aβ formation [20-22]. In the same screening, we found CD74 as an APP-interacting protein as well.

Human CD74 is a type II transmembrane protein of 216 amino acids which functions as a molecular chaperone of class II major histocompatibility complex (MIIC) [23], and at the same time as a receptor for macrophage migration inhibitory factor (MIF), alone [24] or together with CD44 [25]. However, little is known about the relationship between CD74 and APP. Because both BRI2 and BRI3 interact with APP and inhibits APP processing [20-22,26], we investigated the role of CD74 in regulating the processing of APP. Here, we show that CD74 interacts with APP, inhibits Aβ production, and accumulates APP to endocytotic vacuoles produced by CD74.

## Materials and methods

### Split-ubiquitin screening

The construction of APP as bait, and screening method was described elsewhere [20].

### Cell culture, transfection, plasmids, antibodies and Western blotting

Cell lines, transfection methods, mammalian expression constructs of APP, APP-Ncas, APP Swedish, APP London, APP-YFP, myc-tagged Notch, APLP2 were described [20,27]. CD74 is cloned from the two-hybrid screening, and its mouse counterparts, Ii p31 and Ii p41 were generous gifts from Dr. Norbert Koch [28]. All CD74 constructs, both human and mouse, were cloned into pcDNA3.1 vector (Invitrogen) with a N-terminal FLAG tag. The Dutch and Australian mutations of APP were introduced into APP by QuikChange XL (Stratagene).

The following antibodies and antibody beads were used: αFLAG (M2, Sigma F1804); αmyc (Cell Signaling, 2276); αAPP (22C11, Chemicon MAB348); αsAPPα (IBL 11088); αsAPPβ (IBL 18957); αAPP C-terminal fragments (CTF) (αAPPct, Invitrogen/Zymed 36-6900); αAPLP2 (EMD/Calbiochem 171616); α transferrin receptor; Flag M2 beads (Sigma A2220). Rabbit polyclonal antibody and horse radish peroxidase conjugated secondary antibodies are from Southern Biotechnology.

All Western blot samples were separated by 4-12% Criterion Gels (Bio-Rad 3450125) with NuPAGE MES running buffer (Invitrogen NP0002). The gels were transferred to nitrocellulose membrane (Whatman BA85 Protran) with NuPAGE transfer buffer (Invitrogen NP0006) in a Trans-Blot cell (Bio-Rad). After the transfer, the membrane was blocked in PBS containing 5% skim milk, and incubated with indicated primary antibodies over night in the same buffer. The membrane was washed in PBS containing 0.05% Tween-20, and incubated in corresponding secondary antibodies for 1 hour. The membrane was washed in PBS containing 0.05% Tween-20 again, and the bands were visualized with Super Signal West Pico Chemiluminescent Substrate (Pierce) and Hyblot X-ray films (Denville Scientific).

### Immunoprecipitation from HeLa cells

HeLa cells were transfected with indicated combinations of plasmids. The transfected cells were lysed in the Hepes-Triton buffer (20 mM Hepes/NaOH pH 7.4, 1 mM EDTA, 150 mM NaCl, 0.5% Triton-X 100) on ice. The lysates were cleared by spinning at 20,000 g for 10 min. To precipitate the immune complex, the cleared lysates were mixed with FLAG beads, or with indicated antibodies and protein A beads (Pierce). A rabbit polyclonal antibody was used as a negative control of αAPPct precipitation. After being washed three times with the same buffer, the precipitated beads were boiled in 2 × SDS Buffer and analyzed by Westerm blot. The immunoprecipitants analyzed correspond to the four times of the inputs.

### Staining of transfected HeLa cells

HeLa cells were plated on coverslips coated with poly L-lysine (Sigma), and were transfected with indicated plasmids. The cells on coverslips were fixed, permeabilized with 0.2% Triton X-100, stained as described [29]. APP-YFP and Notch-GFP were detected with YFP/GFP fluorescence. Anti-FLAG and αAPPct stainings were visualized with Alexa Fluor 594 goat anti-mouse IgG (H+L) (Invitrogen) and Alexa Fluor 488 goat anti-rabbit IgG (H+L) (Invitrogen), respectively. Cells were imaged sequentially with the Albert Einstein Analytical Imaging Facility BioRad Radiance 2000 confocal microscope using a 63× NA1.4 oil objective and 488 nm and 568 nm laser lines. The images shown are representative of the phenotypes observed in at least three independent experiments.

### Colocalization analysis

Regions of interest were analyzed with ImageJ software (National Institute of Health) and Colocalization Indices plug-in [30]. Colocalization coefficient (CC) [31] and intensity correlation quotient (ICQ) [32] were calculated with the software by setting the threshold of CD74 staining so that the calculation is limited to CD74-positive vacuoles. CC ranges from -1 to 1: 1 indicates complete colocalization; 0, ramdom colocalization, -1, complete negative colocalization. Positive ICQ indicates positive colocalizatioin.

### The measurement of APP fragments of HEK293APP cells

HEK293APP cells were transfected with pcDNA3 or with FLAG-CD74. One day after the transfection, the cells were further incubated in fresh media for 24 hours. The conditioned media were collected and cleared by spinning at 20,000 g for 10 min. Cells were lysed in the Hepes-Triton buffer, and protein concentration was determined by Bradford method using bovine serum albumin as a standard. Aβ in the media and the cell lysates were measured using the ELISA kits for Aβ40 and Aβ42 (IBL 17713 and 17711, respectively).

Equal amounts of total lysates and the cleared media were analyzed by Westerm blot.

### The measurement of stability of synthetic Aβ added to the media

Synthetic Aβ40 and Aβ42 (American Peptide Company) were dissolved in 50 mM Tris/HCl pH 8.0 at 1 mg/ml and stored at -70°C until use. HeLa cells were transfected with pcDNA3 or FLAG-CD74. One day after transfection, the media was replaced with fresh media containing synthetic Aβ40 and Aβ42 at approximately 100 ng/ml. The media were collected at indicated time points and Aβ was measured as above.

### Metabolic labelling of HeLa cells

HeLa cells were transfected with pcDNA3 or FLAG-CD74 together with APP. One day after the transfection, the cells were starved for 90 minutes by incubating in DMEM without cysteine and methionine (Invitrogen). The cells were labelled at 500 Ci/ml of ^35^S cysteine and methionine for 30 minutes and chased in the complete DMEM for indicated times. After the chase, the cells were lysed and immunoprecipitated with αAPPct as above. The precipitants were analyzed by SDS-PAGE and autoradiography.

## Results

### CD74 binds APP

To identify APP ligands, we screened human brain cDNA library with split-ubiquitin system using APP as bait [20]. This screening is designed so that the each bait and prey are fused to each half of modified ubiquitin, which upon interaction releases the DNA binding domain and activation domain. They are translocated to the nuclei and transcriptionally activate the downstream reporter gene [33]. This screening enabled us to clone transmembrane proteins, which are very difficult to clone in regular yeast two hybrid screening because the transmembrane proteins do not translocate to the nucleus to activate the downstream signal.

**Figure 1:**
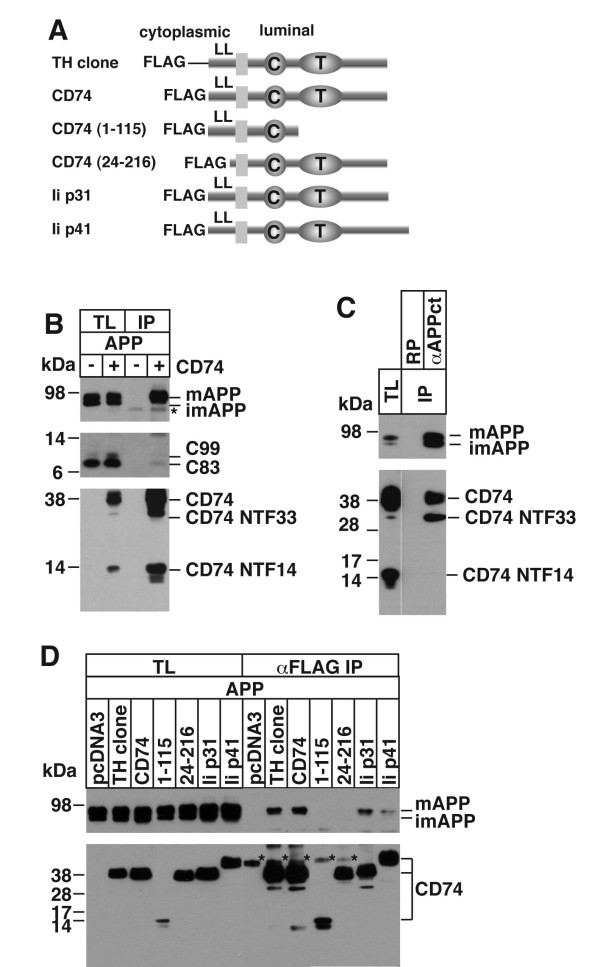
**A) A cartoon of various FLAG-tagged CD74 constructs: the yeast split-ubiquitin (TH) clone, full length CD74, two deletions constructs of CD74, and two splicing variants of mouse CD74**. The cytoplasmic and luminal parts, the locations of the FLAG tag, the endosomal-sorting signal in the cytoplasmic domain (LL), the CLIP domain (C), and the trimeric domain (T) are indicated. B) HeLa cells were transfected with pcDNA3 (-) or FLAG-CD74 (+) together with APP, and the total cell lysates (TL) and FLAG immunoprecipitants (IP) were analyzed by Westerm blot. Full length APP, APP CTF (C99 and C83), and CD74 were detected with 22C11, αAPPct, and αFLAG antibodies, respectively. The 33 and 14 kDa N-terminal fragments of CD74 were designated as CD74 NTF33 and NTF14. C) HeLa cells were transfected with FLAG-CD74 and APP, and the total lysates of the transfected cells were immunoprecipitated with either rabbit polyclonal antibody (RP) or αAPPct, and analyzed as in B. D) HeLa cells were transfected with various CD74 constructs and APP. The total lysates and FLAG IPs were analyzed as in B. Mature APP and immature APP (mAPP/imAPP) are indicated. The bands marked with asterisks (*) were attributed to the FLAG antibody used in the immunoprecipitation.

One of the clones isolated contained the entire CD74 cDNA (Figure 1A). CD74 serves as an invariant chain of Class II major histocompatibility complex (MIIC) [34], and it also serves as a receptor for macrophage migration inhibitory factor [24]. CD74 is a type II transmembrane protein, which is processed by protease during its maturation as a chaperon of Class II MHC molecules [23].

In the same split-ubiquitin yeast screening using APP as bait, we cloned BRI2 and BRI3 [20-22]. Since BRI2 and BRI3 are also single pass, type II transmembrane proteins themselves, and interact with APP and inhibit the production of Aβ [20-22] we decided to test if the same is true for CD74 in mammalian cell context.

Full length CD74 with an N-terminal FLAG tag were transfected to HeLa cells. As shown in Figure 1B, the immunoprecipitation of CD74 from the total lysates indicated that predominantly mature APP (mAPP) interacted with CD74. There is little binding to the two APP C-terminal fragments (APP CTFs, indicated as C99 and C83). No consistent change was observed for the amount of APP CTFs. This CD74-APP interaction was further confirmed by the reciprocal immunoprecipitation. The total lysates were prepared from the HeLa cells cotransfected with FLAG-CD74 and APP, and the CD74 fragments bound to the precipitated APP were analyzed. The Westerm blot shows that CD74 indeed bound to APP. Interestingly, the 33 kDa fragment (CD74 NTF33), but not the 14 kDa fragment (CD74 NTF14) of CD74 was associated with APP, even though NTF14 was clearly the major CD74 NTF in the total lysates (Figure 1B). The preferred binding of NTF33 over NTF14 indicates that NTF 14 is not sufficient for the CD74-APP interaction, and hints that the trimeric region of CD74 is required for the interaction.

Next, we attempted to determine the region of CD74 required for this interaction. cDNA obtained in the two-hybrid screening, full length CD74, two fragments of CD74, and the two mouse CD74 isoforms (p31 and p41) were cloned into a FLAG-tagged mammalian expression vector (Figure 1A), and cotransfected into HeLa cells together with APP. Immunoprecipitation with FLAG beads showed that both human and mouse CD74 bound mature APP. The lack of interaction found in the deletion construct CD74 (1-115) confirms that the luminal portion containing the trimeric domain is required for the CD74 binding to APP. The deletion of first 23 amino acids (CD74 24-216) also disrupted the interaction, hinting to the possibility that the endosomal-targeting signal [35] might be involved in this interaction.

### CD74 does not interact with Notch or APLP2

**Figure 2:**
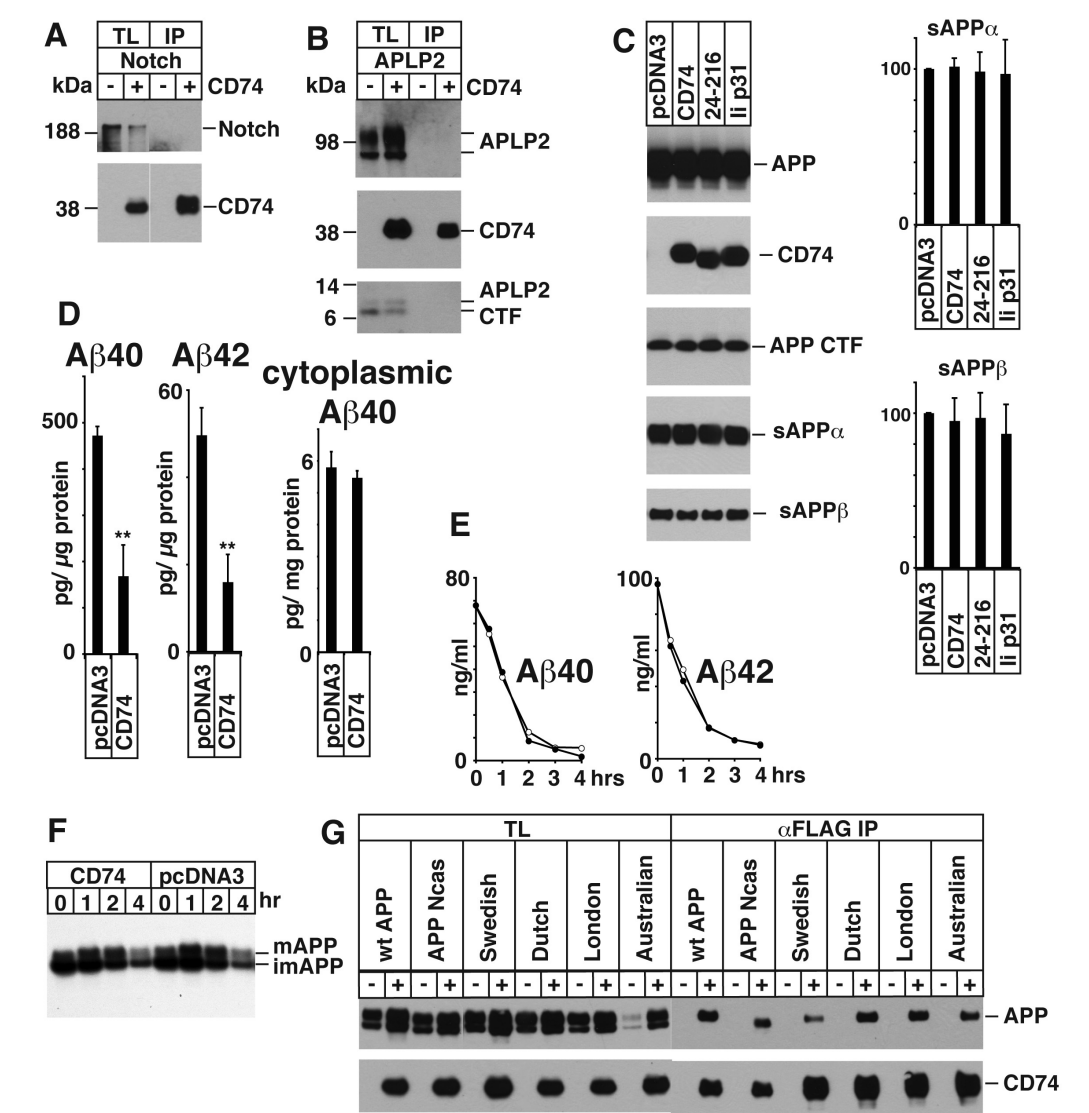
**A) HeLa cells were transfected with pcDNA3 (-) or CD74 (+), together with Notch**. The total cell lysates (TL) and FLAG IPs were analyzed as in Figure 1B. Notch was detected with αmyc antibody. B) HeLa cells were transfected with pcDNA3 (-) or CD74 (+), together with APLP2, and were analyzed as in A. C) Left panels: HEK293APP cells were transfected with the indicated plasmids. The transfected cells were further incubated for 24 hours with fresh media, and secreted sAPPα/β were measured by Westerm blot. The cell lysates were analyzed as in Figure 1B. Right panels: the quantification of sAPPα/β. The levels in pcDNA3 transfected cells were set to 100. D) HEK293APP cells were transfected with pcDNA3 or CD74 and treated as in C. Aβ40 and 42 secreted in the media, and Aβ40 in the lysates were measured with specific ELISA. The amount of Aβ was normalized to the amount of the protein in the total lysates. The asterisks (**) indicate that p values by Student's t test are less than 0.01. E) HeLa cells were transfected with pcDNA3 or CD74, and the media was replaced with fresh media containing synthetic Aβ40 and Aβ42. The media of pcDNA3 (open circles) and CD74 (closed circles) transfected cells were incubated for indicated hours, and the remaining Aβ40 and Aβ42 were measured as in D. F) HeLa cells were transfected with pcDNA3 or FLAG-CD74 together with APP. The cells were pulsed for 30 min with ^35^S methionine+cysteine, and chased in fresh media for indicated hours. APP was immunoprecipitated with αAPPct antibody and analyzed by SDS-PAGE followed by autoradiography. G) HeLa cells were transfected with pcDNA3 (-) or FLAG-CD74 (+) together with wild type APP (wt APP) or the indicated mutants. The total lysates and FLAG IPs were processed as in Figure 1B. Note that APP Ncas lacks C-terminal 31 amino acids of APP and has therefore a smaller in size.

To investigate the specificity of CD74-APP binding, myc-tagged Notch and APLP2 were transfected into HeLa cells together with pcDNA3 or FLAG-CD74. They lysates from the transfected cells were precipitated with FLAG beads to analyze if Notch or APLP2 co purified with CD74. As indicated in Figure 2A, CD74 did not precipitate Notch. Nor did it precipitate APLP2, a close homolog of APP (Figure 2B). The lack of interaction with Notch or APLP2 further attests the specificity of the CD74-APP interaction.

### CD74 over expression does not change sAPPα and sAPPβ

Next, we tested how other APP metabolites were affected by the overexpression of CD74. HeLa cells were transfected with APP together with pcDNA3, FLAG-CD74, FLAG-CD74 (24-216), and mouse CD74 (Ii p31), and one day later, the cells were further incubated in fresh media for 24 hours. APP fragments in the media and in the cell were analyzed by Westerm blot. As shown in Figure 2C, CD74 over expression did not show significant change in APP, APP CTFs, sAPPα, or sAPPβ (left panels: Western blots, right panels: quantification of the blots). Student's t test showed no significant difference between any combinations of the values.

### CD74 over expression reduces secreted Aβ, but does not change the cytoplasmic Aβ40

Next, we asked if CD74 changes the production of Aβ. HEK293APP cells, which stably express APP, were transfected with pcDNA3 or with FLAG-CD74. The transfectants were further incubated in fresh media as above and the secreted Aβ40 and Aβ42 were measured with Aβ ELISA. As shown in Figure 2D, exogenous expression of CD74 decreased both the secreted Aβ40 and Aβ42. On the contrary, there is no significant change of cytoplasmic Aβ40 (Figure 2E). The levels of cytoplasmic Aβ42 were too low to be detected (data not shown).

### CD74 overexpression does not change the stability of Aβ added to the media, or the metabolism of cell-bound APP

Next, we questioned if the overexpression of CD74 affects the stability of Aβ added to the media. HeLa cells were transfected with pcDNA3 or FLAG-CD74. On the next day, the media was replaced with fresh media containing of Aβ40 and Aβ42. The ELISA of the media taken at the indicated time points showed there was no significant difference in the stability of Aβ40 (p = 0.890) and Aβ42 (p = 0.141) by paired Student's t test analysis (Figure 2E).

In order to determine whether metabolism of APP is modified by CD74, we performed pulse-chase labeling of APP. HeLa cells were transfected with pcDNA3 or FLAG-CD74 together with APP. The cells were pulse labeled with ^35^S methionine and cysteine and chased for indicated hours. The autoradiography of immunoprecipitated APP indicates that CD74 did not change the conversion of cell-bound APP significantly (Figure 2F).

### CD74 interacts with APP-Ncas, and other APP FAD mutants

Next, we tested if various mutants of APP interact with CD74. HeLa cells were transfected with APP, APP-Ncas, APP Swedish, APP Dutch, APP London, and APP Australian mutants. These are all APP mutations that in humans cause familial forms of Alzheimer's disease [36], except for APP-Ncas that is an APP construct missing the COOH-terminal 31 amino acids [37]. The lysates were prepared from the transfected cells, and CD74 was precipitated. The FLAG immunoprecipitations show that CD74 fully interacts with mature form of all APP variants (Figure 2G).

### CD74 changes the intracellular distribution of APP

**Figure 3:**
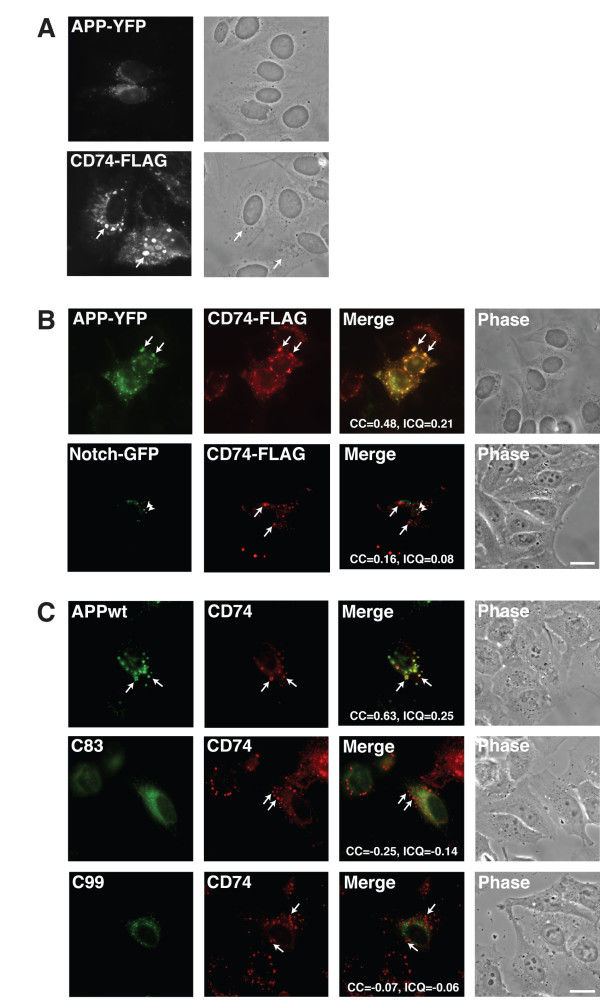
**A) HeLa cells were transfected with APP-YFP or FLAG-CD74**. APP and CD74 were detected by YFP fluorescence and αFLAG antibody staining with Alexa594 anti-mouse secondary antibody, respectively. Corresponding phase contrast views were shown. B) HeLa cells were transfected with APP-YFP or Notch-GFP together with FLAG-CD74. Notch was detected by GFP fluorescence. APP and CD74 were detected as in A. Digitally merged pictures of Green and Red channels and phase contrast view are also displayed. Colocalization Coefficient (CC) and Intensity Correlation Quotient (ICQ) were indicated. C) HeLa cells were transfected with APP, C99 or C83 together with FLAG-CD74. APP, C99, and C83 were detected with the αAPPct antibody, and FLAG-CD74 was detected as above. Merged pictures and phase contrast views were shown as in B. Bar = 10 μm. CC and ICQ are as in B.

Next, we sought to identify how CD74 and APP are distributed inside the cell. As shown in Figure 3A for Hela cells transfected with APP-YFP or FLAG-CD74, APP is localized in intracellular reticular structures, while CD74 is targeted to endosomes [35]. Overexpression of CD74 and has been reported to create vacuoles [35] that we also can appreciate (Figure 3A).

When these two were co expressed in the same cell, a significant portion of APP co localized with CD74 in these vacuoles (Figure 3B, upper panels). This CD74-dependent redistribution of APP is specific since, for example, CD74 did not co-localize in these vacuolar structures with Notch-GFP, reinforcing the above mentioned biochemical observation that CD74 does not bind Notch (Figure 2A).

Next, we tested if the major APP CTF fragments, C99 and C83, changed intracellular localization if expressed together with CD74. For this, HeLa cells were cotransfected with these APP variants and FLAG-CD74, and stained with αAPPct to visualize APP, C99 and C83, and αFLAG to visualize CD74. As expected, APP co localized with CD74 nicely in the vacuolar structures. However, C99 and C83 did not show detectable accumulation to the CD74 induced vacuolar structures. This observation could explain the initial immunoprecipitations results that CD74 barely interact with APP CTFs (Figure 1B).

## Discussion

We isolated CD74, together with the familial dementia gene BRI2 and its family member BRI3, during a genetic screening for membrane-bound APP ligands [20,21]. CD74 is known to work as a chaperon of class II major histocompatibility complex in B cells, which present antigens to T cells [34,38]. Interestingly, the functions of CD74 are not limited to the chaperons in the immune cells. Recently, CD74 has been proposed to serve as a receptor for macrophage migration inhibitory factor [24].

CD74 associates with Class II MHC in endoplasmic reticulum. After it is transported through post-Golgi compartments, the complex are diverted to endocytic vesicles, and CD74 is proteolytically processed to CLIP region (Figure 1A), which remains bound to Class II MHC [23,38]. APP does not associate CD74 in endoplasmic reticulum, since only fully glycosylated APP binds to CD74 (Figure 1B), and CD74 does not change the rate of maturation of APP (Figure 2F). Thus it is unlikely that CD74 functions as ER chaperones as in the case of Class II MHC.

We previously showed that the familial dementia gene BRI2 and its homologue BRI3 are inhibitors of APP processing [20,21]. Here, we have established that CD74 binds APP and inhibits the production of Aβ. Like the two members of the *BRI *gene family, CD74 is also a type II transmembrane protein, but structurally unrelated to BRIs (Figure 1A). CD74 can immunoprecipitate APP (Figure 1B). Reciprocal immunoprecipitations shows APP can precipitate CD74 (Figure 1C). Both N-terminal 23 amino acids and C-terminal 101 amino acids of CD74 are required for the interaction (Figure 1D). On the APP side, C99 and C83 scarcely bind CD74 (Figure 1A).

This binding of CD74 to APP is similar to that of BRI2 or BRI3, in that only the mature form of APP binds CD74 (Figure 1A). However, there is a clear difference: CD74 does not cause the accumulation of C99 as in the case of BRI2, nor does it bind to C99 (Figure 1B). Or, contrary to BRI2 and BRI3, CD74 does not inhibit secretion of sAPPα or sAPPβ (Figure 2C) [20-22]. Therefore, it is unlikely that CD74 is working on APP by masking the secretase sites as in the case of BRI2 and BRI3. Rather, CD74 might be redirecting APP to intracellular compartments where the production of Aβ in reduced.

As shown in Figure 2D-F, CD74 does reduce the Aβ in the media when APP is co transfected, but it does not change the cytoplasmic Aβ40 (Figure 2D), or the stability of exogenously added Aβ (Figure 2E), or the maturation of APP (Figure 2F). These data further support our notion that CD74 works on APP inside cells after APP is matured, and not by destabilizing Aβ already secreted in the media.

It is noteworthy that the major metabolite of CD74 (CD74NTF14) does not interact with APP, but the minor fragment (NTF33) does (Figure 1C). This observation strongly argues that the CD74-APP interaction is specific because APP distinguishes NTF33 from NTF14; and the interaction occurs before CD74 is irreversibly processed further than NTF33; and at least NTF33 is required for the interaction. We cannot conclude that NTF33 is the minimal region required for the CD74-APP interaction because NTF33 might interact with APP indirectly, in a trimer with full length CD74. The precise dissection of the minimal region responsible for the binding requires further study, and should take into account the fact that CD74 forms trimers [34,38].

APP and CD74 displays distinct intracellular distribution. When transfected independently, APP displays reticular and vesicular structures in the cytoplasm. In contrast, CD74 shows larger vacuoles, which are endosomal in nature [35]. Our observation that APP co localizes with CD74 in vacuolar structure in HeLa cells (Figure 3B, upper panels) supports the notion that CD74 and APP interact. Moreover, the striking APP re-distribution to CD74-positive vacuolar structures suggests that this change is caused by the physical interaction of CD74 and APP. Co expression of CD74 and either Notch, C99 or C83 (all molecules that do not bind CD74) show the specificity of this phenomenon since Notch, C99 and C83 do not accumulate in CD74-positive vacuoles upon co expression (Figure 3B, lower panels, and 3C).

Thus we propose that unlike BRI2 or BRI3, which are working directly on APP by blocking the access of secretases to APP, CD74 interferes with APP processing by regulating the sub-cellular localization of APP in compartments where amyloidogenic APP cleavage is reduced.

Little is known about the relationship between CD74 and AD. In immuno-histochemistry experiments, normal brains do not stain well for CD74 and CD74 is mostly expressed in microglia. However, the brains of AD patients have increased CD74 staining [39]. Interestingly, a recent study shows that CD74 is accumulated in neurons with paired helical fibers [40]. Our findings suggest that CD74 may be a player in controlling APP biology and processing, perhaps regulating APP trafficking in neurons. Given the anti-amyloidogenic activity of CD74, it is possible that the increased levels of CD74 in AD brains, potentially caused by increased TNFα in AD patients and in transgenic mouse over expressing APP [41-43], could contribute to a negative feedback mechanism of the organism to fend off noxious APP processing up regulation.

## Competing interests

The authors declare that they have no competing interests.

## Authors' contributions

SM conceived of the study, carried out yeast and biochemical studies and drafted the manuscript. YM carried out the molecular biological work and immunocytochemical analysis. LD conceived the study, participated in the design and coordination. All authors read and approved the final manuscript.

## References

[B1] Breteler MM, Claus JJ, van Duijn CM, Launer LJ, Hofman A (1992). Epidemiology of Alzheimer's disease. Epidemiol Rev.

[B2] Selkoe DJ, Podlisny MB (2002). Deciphering the genetic basis of Alzheimer's disease. Annu Rev Genomics Hum Genet.

[B3] Selkoe D, Kopan R (2003). Notch and Presenilin: regulated intramembrane proteolysis links development and degeneration. Annu Rev Neurosci.

[B4] Sisodia SS, St George-Hyslop PH (2002). gamma-Secretase, Notch, Abeta and Alzheimer's disease: where do the presenilins fit in?. Nat Rev Neurosci.

[B5] Hamid R, Kilger E, Willem M, Vassallo N, Kostka M, Bornhovd C, Reichert AS, Kretzschmar HA, Haass C, Herms J (2007). Amyloid precursor protein intracellular domain modulates cellular calcium homeostasis and ATP content. J Neurochem.

[B6] Madeira A, Pommet JM, Prochiantz A, Allinquant B (2005). SET protein (TAF1beta, I2PP2A) is involved in neuronal apoptosis induced by an amyloid precursor protein cytoplasmic subdomain. FASEB J.

[B7] Passer B, Pellegrini L, Russo C, Siegel RM, Lenardo MJ, Schettini G, Bachmann M, Tabaton M, D'Adamio L (2000). Generation of an apoptotic intracellular peptide by gamma-secretase cleavage of Alzheimer's amyloid beta protein precursor. J Alzheimers Dis.

[B8] Cao X, Sudhof TC (2001). A transcriptionally [correction of transcriptively] active complex of APP with Fe65 and histone acetyltransferase Tip60. Science.

[B9] Cupers P, Orlans I, Craessaerts K, Annaert W, De Strooper B (2001). The amyloid precursor protein (APP)-cytoplasmic fragment generated by gamma-secretase is rapidly degraded but distributes partially in a nuclear fraction of neurones in culture. J Neurochem.

[B10] Pardossi-Piquard R, Petit A, Kawarai T, Sunyach C, Alves da Costa C, Vincent B, Ring S, D'Adamio L, Shen J, Muller U (2005). Presenilin-dependent transcriptional control of the Abeta-degrading enzyme neprilysin by intracellular domains of betaAPP and APLP. Neuron.

[B11] Liu Q, Zerbinatti CV, Zhang J, Hoe HS, Wang B, Cole SL, Herz J, Muglia L, Bu G (2007). Amyloid precursor protein regulates brain apolipoprotein E and cholesterol metabolism through lipoprotein receptor LRP1. Neuron.

[B12] von Rotz RC, Kohli BM, Bosset J, Meier M, Suzuki T, Nitsch RM, Konietzko U (2004). The APP intracellular domain forms nuclear multiprotein complexes and regulates the transcription of its own precursor. J Cell Sci.

[B13] Kim HS, Kim EM, Lee JP, Park CH, Kim S, Seo JH, Chang KA, Yu E, Jeong SJ, Chong YH, Suh YH (2003). C-terminal fragments of amyloid precursor protein exert neurotoxicity by inducing glycogen synthase kinase-3beta expression. FASEB J.

[B14] Baek SH, Ohgi KA, Rose DW, Koo EH, Glass CK, Rosenfeld MG (2002). Exchange of N-CoR corepressor and Tip60 coactivator complexes links gene expression by NF-kappaB and beta-amyloid precursor protein. Cell.

[B15] Checler F, Sunyach C, Pardossi-Piquard R, Sevalle J, Vincent B, Kawarai T, Girardot N, St George-Hyslop P, da Costa CA (2007). The gamma/epsilon-secretase-derived APP intracellular domain fragments regulate p53. Curr Alzheimer Res.

[B16] Leissring MA, Murphy MP, Mead TR, Akbari Y, Sugarman MC, Jannatipour M, Anliker B, Muller U, Saftig P, De Strooper B (2002). A physiologic signaling role for the gamma-secretase-derived intracellular fragment of APP. Proc Natl Acad Sci USA.

[B17] Giliberto L, Zhou D, Weldon R, Tamagno E, De Luca P, Tabaton M, D'Adamio L (2008). Evidence that the Amyloid beta Precursor Protein-intracellular domain lowers the stress threshold of neurons and has a "regulated" transcriptional role. Mol Neurodegener.

[B18] Scheinfeld MH, Matsuda S, D'Adamio L (2003). JNK-interacting protein-1 promotes transcription of A beta protein precursor but not A beta precursor-like proteins, mechanistically different than Fe65. Proc Natl Acad Sci USA.

[B19] Roncarati R, Sestan N, Scheinfeld MH, Berechid BE, Lopez PA, Meucci O, McGlade JC, Rakic P, D'Adamio L (2002). The gamma-secretase-generated intracellular domain of beta-amyloid precursor protein binds Numb and inhibits Notch signaling. Proc Natl Acad Sci USA.

[B20] Matsuda S, Giliberto L, Matsuda Y, Davies P, McGowan E, Pickford F, Ghiso J, Frangione B, D'Adamio L (2005). The familial dementia BRI2 gene binds the Alzheimer gene amyloid-beta precursor protein and inhibits amyloid-beta production. J Biol Chem.

[B21] Matsuda S, Giliberto L, Matsuda Y, McGowan EM, D'Adamio L (2008). BRI2 inhibits amyloid beta-peptide precursor protein processing by interfering with the docking of secretases to the substrate. J Neurosci.

[B22] Matsuda S, Matsuda Y, D'Adamio L (2009). BRI3 inhibits amyloid precursor protein processing in a mechanistically distinct manner from its homologue dementia gene BRI2. J Biol Chem.

[B23] Matza D, Kerem A, Shachar I (2003). Invariant chain, a chain of command. Trends Immunol.

[B24] Leng L, Metz CN, Fang Y, Xu J, Donnelly S, Baugh J, Delohery T, Chen Y, Mitchell RA, Bucala R (2003). MIF signal transduction initiated by binding to CD74. J Exp Med.

[B25] Shi X, Leng L, Wang T, Wang W, Du X, Li J, McDonald C, Chen Z, Murphy JW, Lolis E (2006). CD44 is the signaling component of the macrophage migration inhibitory factor-CD74 receptor complex. Immunity.

[B26] Fotinopoulou A, Tsachaki M, Vlavaki M, Poulopoulos A, Rostagno A, Frangione B, Ghiso J, Efthimiopoulos S (2005). BRI2 interacts with amyloid precursor protein (APP) and regulates amyloid beta (Abeta) production. J Biol Chem.

[B27] Matsuda S, Matsuda Y, D'Adamio L (2003). Amyloid beta protein precursor (AbetaPP), but not AbetaPP-like protein 2, is bridged to the kinesin light chain by the scaffold protein JNK-interacting protein 1. J Biol Chem.

[B28] Koch N, Lauer W, Habicht J, Dobberstein B (1987). Primary structure of the gene for the murine Ia antigen-associated invariant chains (Ii). An alternatively spliced exon encodes a cysteine-rich domain highly homologous to a repetitive sequence of thyroglobulin. EMBO J.

[B29] Matsuda S, Yasukawa T, Homma Y, Ito Y, Niikura T, Hiraki T, Hirai S, Ohno S, Kita Y, Kawasumi M (2001). c-Jun N-terminal kinase (JNK)-interacting protein-1b/islet-brain-1 scaffolds Alzheimer's amyloid precursor protein with JNK. J Neurosci.

[B30] Nakamura K, Watakabe A, Hioki H, Fujiyama F, Tanaka Y, Yamamori T, Kaneko T (2007). Transiently increased colocalization of vesicular glutamate transporters 1 and 2 at single axon terminals during postnatal development of mouse neocortex: a quantitative analysis with correlation coefficient. Eur J Neurosci.

[B31] Manders EM, Stap J, Brakenhoff GJ, van Driel R, Aten JA (1992). Dynamics of three-dimensional replication patterns during the S-phase, analysed by double labelling of DNA and confocal microscopy. J Cell Sci.

[B32] Li Q, Lau A, Morris TJ, Guo L, Fordyce CB, Stanley EF (2004). A syntaxin 1, Galpha(o), and N-type calcium channel complex at a presynaptic nerve terminal: analysis by quantitative immunocolocalization. J Neurosci.

[B33] Stagljar I, Korostensky C, Johnsson N, te Heesen S (1998). A genetic system based on split-ubiquitin for the analysis of interactions between membrane proteins in vivo. Proc Natl Acad Sci USA.

[B34] Cresswell P (1994). Antigen presentation. Getting peptides into MHC class II molecules. Curr Biol.

[B35] Bakke O, Dobberstein B (1990). MHC class II-associated invariant chain contains a sorting signal for endosomal compartments. Cell.

[B36] Price DL, Sisodia SS (1998). Mutant genes in familial Alzheimer's disease and transgenic models. Annu Rev Neurosci.

[B37] Pellegrini L, Passer BJ, Tabaton M, Ganjei JK, D'Adamio L (1999). Alternative, non-secretase processing of Alzheimer's beta-amyloid precursor protein during apoptosis by caspase-6 and -8. J Biol Chem.

[B38] Cresswell P (1994). Assembly, transport, and function of MHC class II molecules. Annu Rev Immunol.

[B39] Yoshiyama Y, Arai K, Oki T, Hattori T (2000). Expression of invariant chain and pro-cathepsin L in Alzheimer's brain. Neurosci Lett.

[B40] Bryan KJ, Zhu X, Harris PL, Perry G, Castellani RJ, Smith MA, Casadesus G (2008). Expression of CD74 is increased in neurofibrillary tangles in Alzheimer's disease. Mol Neurodegener.

[B41] Cunningham C, Wilcockson DC, Campion S, Lunnon K, Perry VH (2005). Central and systemic endotoxin challenges exacerbate the local inflammatory response and increase neuronal death during chronic neurodegeneration. J Neurosci.

[B42] Holmes C, Cunningham C, Zotova E, Woolford J, Dean C, Kerr S, Culliford D, Perry VH (2009). Systemic inflammation and disease progression in Alzheimer disease. Neurology.

[B43] Popp J, Bacher M, Kolsch H, Noelker C, Deuster O, Dodel R, Jessen F (2009). Macrophage migration inhibitory factor in mild cognitive impairment and Alzheimer's disease. J Psychiatr Res.

